# A pH-responsive complex based on supramolecular organic framework for drug-resistant breast cancer therapy

**DOI:** 10.1080/10717544.2021.2021325

**Published:** 2021-12-30

**Authors:** Yun-Chang Zhang, Pei-Yu Zeng, Zhi-Qiang Ma, Zi-Yue Xu, Ze-Kun Wang, Beibei Guo, Feng Yang, Zhan-Ting Li

**Affiliations:** a School of Pharmacy, Naval Medical University, Shanghai, China; b Department of Chemistry, Shanghai Key Laboratory of Molecular Catalysis and Innovative Materials, Fudan University, Shanghai, China

**Keywords:** Supramolecular organic framework, drug delivery system, doxorubicin, drug resistance, breast cancer

## Abstract

Chemotherapy is one of the main ways to treat breast cancer clinically. However, the multidrug resistance to anti-tumor drugs limits their clinical use. To overcome these drawbacks, development of drug delivery systems (DDSs) has attracted more and more attention in cancer therapy. At present, the preparation and purification process are complicated for many reported DDSs, while clinic calls for new DDSs that are more convenient for preparation. Here, a new pH-responsive supramolecular organic framework drug delivery complex loading doxorubicin (DOX) is fabricated. Anti-tumor activity of the system *in vitro* was investigated by cell cytotoxicity, uptake assay, and cell apoptosis analysis. The anti-tumor activity *in vivo* was investigated by inspecting nude mice body weight, tumor volume, and weight, also a preliminary mechanism probe was conducted by HE and TUNEL staining. The DOX@SOF displayed high stability, good biocompatibility, and pH regulated drug release. At acid condition, the hydrazone bonds would be broken, which result in the dissociation of SOF, and then the drugs would be released from the system. Furthermore, DOX@SOF enhanced cellular internalization. Both *in vitro* and *in vivo* experiments reflected that DOX@SOF could enhance the anti-tumor activity of DOX for the MCF-7/ADR tumor cells and tumors. This study provides a highly efficient strategy to prepare stimulus-responsive supramolecular drug delivery complex for treatment of drug-resistant cancer, the results presented inspiring scientific interests in exploring new drug delivery strategy and reversing multi-drug resistance for clinical chemotherapy.

## 1. Introduction

By 2020, for the first time breast cancer overtook lung cancer and had become the most common cancer in the world, its incidence and death is the first health threat in women (Siegel et al., 2020). Chemotherapy is one of the most widely utilized and effective methods in clinical treatment of breast cancer (Liyanage et al., 2019). Chemotherapy refers to the application of anti-tumor drugs (doxorubicin (DOX), docetaxel, paclitaxel, tamoxifen, etc.) to patients alone or in combination (Poustchi et al., 2021). Doxorubicin, also known as adriamycin, is a first-line chemotherapy drug for the treatment of breast cancer (Di et al., 2016; Luo et al., 2020). The main mechanism of is that the quinone-hydro-quinone structure on the anthracycline can be inserted between adjacent base pairs of DNA, destructing the DNA structure, interfering with transcription, and inhibiting mRNA synthesis (Wang et al., 2014; Sun et al., 2019). Although DOX has been in clinical cancer treatment, it further application is strictly confined as bone marrow suppression, cardiotoxic side effects, and dose-dependent cardiotoxicity (Lovitt et al., 2018). More seriously, multidrug resistance has become a huge barrier to limit its clinical use (Li et al., 2020; Xu et al., 2020; You et al., 2021). Researchers have proposed various mechanisms that may explain the multidrug resistance of tumor cells (Lv et al., 2014; Niraula & Ocana, 2016; Ding et al., 2017; Luo et al., 2018; Li & Li, 2021). P-glycoprotein (P-gp), an important efflux transporter with ‘drug pump’ function, can bind to drug molecules and pump drug molecules out of tumor cells, reducing the concentration of drugs in tumor cells to induce multidrug resistance of tumor cells, which is one of the most important mechanisms for tumor cells to produce multidrug resistance (Wu et al., 2016; Ge et al., 2017). Studies have found that nanoparticles can enter cells through endocytosis, avoiding being excreted by P-gp (Chen et al., 2015; Wang et al., 2019). Therefore, in order to reduce the toxic and side effects of chemotherapeutic drugs and overcome the multidrug resistance of tumor cells, it is necessary to construct new drug delivery systems (DDSs) with high drug load that can actively target tumor tissues and regulate drug release.

In recent decades, supramolecular chemistry and self-assembly strategies have attracted more and more attention (Liu et al., 2012; Zhao et al., 2014; Jiang et al., 2016; Ashwanikumar et al., 2018; Sun et al., 2018; Zhang & Zhang, 2019; Li et al., 2021a, 2021b; Qin et al., 2021; Zhang et al., 2021). Supramolecular systems have multifunctional and dynamic regulation properties, and supramolecular components can reversibly change shape and structure according to changes in the external environment to control the release of embedded drugs (Stoffelen & Huskens, 2015; Putaux et al., 2017; Yang et al., 2018; Liu et al., 2021). Thus, supramolecular self-assembly has become a potential strategy for the development of new drug delivery methods. Recently, our team has developed a three-dimensional supramolecular organic frameworks in aqueous atmosphere with self-assembly strategy, which utilized hydrophobically driven encapsulation of the dimers formed by aromatic units by the cucurbit[8]uril (CB[8]) ring (Yang et al., 2018). In particular, the SOFs with well-defined pores have great potential in adsorbing and releasing drugs (Tian et al., 2016, 2017; Yao et al., 2017). In order to achieve efficient and controlled release, various stimuli-responsive (such as pH) techniques have been developed in the past decade (Zhang et al., 2019a, 2019b, 2020; Zhu et al., 2020; Li et al., 2021c). Therefore, it is of great scientific and clinical interest to explore new responsive SOFs for the construction of DDSs.

**Scheme 1.**
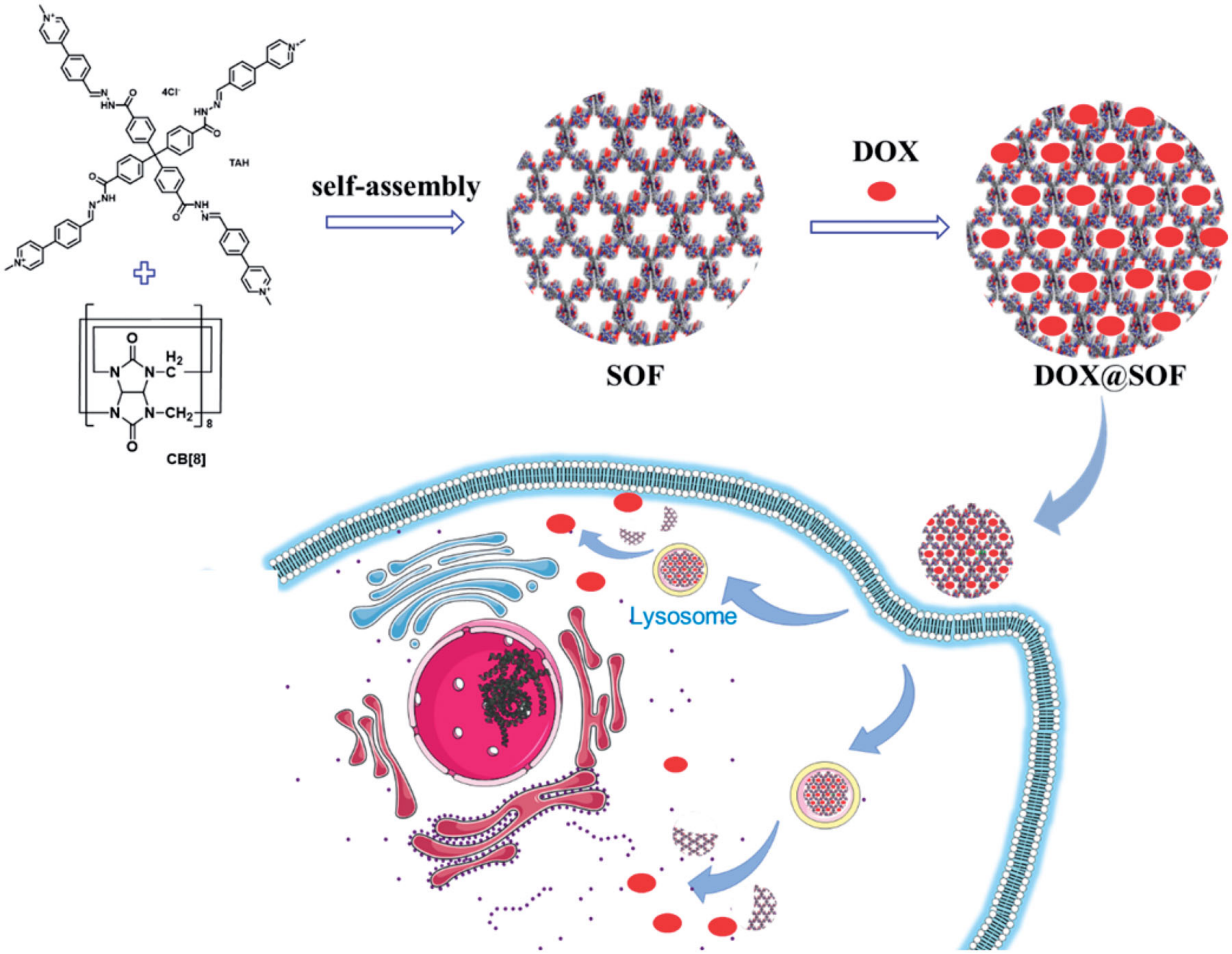
Illustration of the preparation of the drug delivery system DOX@SOF and the proposed model for acid-activable drug release in tumor cells.

Herein, we fabricated a pH-responsive three-dimensional supramolecular organic framework utilizing hydrophobically driven encapsulation of the dimers of aromatic units by the CB[8] ring for the first time, in which the hydrazone bonds in the tetrahedral monomer endows the SOF a good pH responsiveness. The SOF not only maintains the respective structural characteristics of the original single molecular components, but also demonstrates the profiles of the new self-assembled structure. The SOF with the highly hydrophobic nature of their pores could physically envelop DOX through hydrophobic interaction and electrostatic interaction. Compared with a large number of other DDS, DOX@SOF can be simply prepared by dispersing DOX and SOF *in situ* to self-assembly into nano complex, which greatly simplifies the widely studied nano-drug carrier systems. Our data demonstrate that the DOX@SOF system can overcome the multidrug resistance of human breast cancer MCF-7/Adr tumor cells *in vitro*, and effectively inhibit the growth of MCF-7/Adr tumor *in vivo* (Scheme 1).

## 2. Material

4,4′,4″,4′-Methyltetrabenzoic acid, methyl iodide, tetrabutylammonium chloride, ammonium hexafluorophosphate, hydrazine hydrate, 4′,6-diamino-2-phenylindole dihydrochloride (DAPI), Lysotracker Green DND-26 were purchased from Shanghai Aladdin Biochemical Technology Co., Ltd. (Shanghai, China), methanol, acetonitrile, and acetone were purchased from Shanghai Titan Technology Co., Ltd. (Shanghai, China), CCK-8 was purchased from Dojindo Laboratories (Kumamoto, Japan), and adriamycin hydrochloride was purchased from Dalian Meilun Biotechnology Co., Ltd. (Dalian, China). All commercial reagents were used without further purification.

## 3. Methods

### 3.1. Preparation of SOF

Before preparation of SOF, a mixture of 4,4′,4″,4‴-methanetetrayltetra(benzohydrazide) (0.55 g, 1.0 mmol) and 4-(1-methyl-pyridin-4-yl)benzaldehyde, chloride salt (0.93 g, 4.0 mmol) in water (20 mL) was stirred at reflux for eight hours and then cooled to room temperature. Acetone was added to the solution until no precipitate was formed. The precipitate was filtrated and washed with ethanol (20 mL) and diethyl ether (20 mL), and then dried in vacuum to afford the TAH 1.3 g as a light yellow solid (yield 95%). The self-assembly of a supramolecular organic framework was achieved from compound TAH and CB[8] (molar ratio 1:2) in water through the encapsulation of the CB[8] macrocycles for the intermolecular dimers formed by the appended aromatic arms (PhPy units) of TAH.

### 3.2. Characterization of SOF

The hydrodynamic diameter (*D*_H_) of SOF at different concentrations in water was measured by dynamic light scattering instrument. Transmission electron microscopy (TEM) was used to obtain the morphology, elementary composition, and diffractive imaging of SOF. Synchrotron small-angle X-ray scattering (SAXS) experiments were performed to determine the periodicity of SOF in solution phase.

### 3.3. Preparation of DOX@SOF

Sixty milligrams of SOF and 4 mg of DOX were dissolved in 8 mL of water, the mixture was stirred for four hours, then put into a dialysis bag (MWCO 1000 Da) immerged in the PBS (1 M) buffers for dialysis (change the buffers every 4–6 hours). When the absorbance of the drug in the buffers became almost undetectable by UV–vis spectroscopy (absorbancy < 0.003), the system was dried with a freeze dryer to obtain DOX@SOF powder for later use. The amount of DOX that loaded into the SOF was determined by UV–vis spectroscopy. The DOX loading efficiency (DLE) was calculated by the formula:

$$\text{DLE}\;\text{(\%)=}\frac{\text{amount of DOX entrapped in SOF}}{\text{initial amount of DOX added}}\times 100\%$$

### 3.4. DOX release *in vitro*

DOX release from DOX@SOF was investigated in different conditions. DOX@SOF was dialyzed against buffer solution of pH 7.4, 5.6, and 4.5 to evaluate the influence of pH values on drug release. Briefly, 38 mg of freeze-dried DOX@SOF dispersed in 4 mL deionized water was added into a dialysis bag (MWCO 1000 Da). Then, three dialysis bags in three glass bottles that containing 30 mL buffers of different pH values were vortexed (200 rpm) at 37 °C. At each time, 2 mL of the release medium was collected and equivalent fresh buffer solution was replenished. Finally, the amounts of DOX release were evaluated by UV–vis spectroscopy.

### 3.5. Cell culture assay

RPMI-1640 medium and PBS were obtained from HyClone (Logan, UT). Fetal bovine serum was purchased from Gibco (Shanghai, China). Trypsin EDTA solution and penicillin–streptomycin solution were purchased from Boguang Biotechnology Co., Ltd. (Shanghai, China). Human breast cancer cells (MCF-7 and MCF-7/ADR) were obtained from Fudan University (Shanghai, China). Human breast cancer cells (MCF-7 and MCF-7/ADR) were cultured in RPMI-1640 with 10% FBS, 1% penicillin, and 1% streptomycin in an incubator with a humidified atmosphere of 5% CO_2_ at 37 °C.

### 3.6. Cytotoxicity, apoptosis assay

The cytotoxicity of SOF and DOX@SOF was detected by CCK-8 assay. These cells were seeded in 96-well plates and cultivated for 12 hours. Then, free drugs and DOX@SOF were added at different concentrations for 48 hours. Finally, complete medium was removed and washed by PBS (1 M), and 100 μL fresh complete medium containing 10% CCK-8 was added. The viability of cells was detected by a microplate reader (EL × 800; Bio Tek, Winooski, VT). The MCF-7/ADR cells were seeded in six-well plates at a density of 3 × 10^5^ cells per well for 12 hours, and then treated with SOF, DOX, and DOX@SOF for 24 hours. Then, the cells and the suspension were collected and stained with the FITC Annexin V-APC/PI Kit (Shanghai Biotend Technology Co. Ltd., Shanghai, China). Finally, apoptosis of the cells was detected and analyzed by flow cytometry.

### 3.7. Cellular uptake assays

The MCF-7/ADR cells were seeded in six-well plates at a density of 3 × 10^5^ cells per well for 12 hours, and then treated with DOX and DOX@SOF (4 µg/mL DOX) for six hours. Then, the cells were washed with PBS three times and harvested in trypsin without EDTA. Finally, the cells were collected by centrifugation at 1000 rpm for five minutes at 37 °C and resuspended in 0.3 mL PBS. Flow cytometry was performed to analyze cell uptake.

Additionally, cellular uptake was also evaluated by confocal laser scanning microscopy (CLSM). The MCF-7/ADR cells were seeded in confocal plates at a density of 8 × 10^3^ cells for 12 hours, and treated with DOX and DOX@SOF (4 µg/mL DOX) for six hours. The cells were washed with PBS three times and pretreated with Lyso Tracker Green DND-26 (750 ng/mL) for one hour. Then, the cells were fixed in 500 µL 4% paraformaldehyde for 20 min at room temperature. Again, the cells were washed with PBS three times and pretreated with 500 µL DAPI for 5 min. Finally, all the cells were visualized on LSCM.

### 3.8. Animals model

Female BABL/c nude mice (7 weeks old, average body weight 17–20 g) were purchased from the Animal Experimental Center of Naval Medical University (Shanghai, China). Nude mice were feed in a specific pathogen free (SPF) animal laboratory. MCF-7/ADR cells (5 × 10^6^ cells per mouse) were injected subcutaneously into the left armpit of nude mice to generate the MCF-7/ADR mouse model. All animals received care in compliance with the guidelines outlined in the Guide for the Care and Use of Laboratory Animals. All procedures were approved by the Institutional Animal Care and Use Committee of the Naval Medical University.

### 3.9. *In vivo* antitumor efficiency

When the tumor volume of the nude mice reached approximately 30 mm^3^, the nude mice were randomly divided into four groups: PBS (control), DOX, SOF, and DOX@SOF group. Drugs were injected via the tail vein every other day with a dosage of 4.5 mg/kg (equivalent concentration of DOX). The body weights and tumor volumes of nude mice were recorded every other day. The tumor volumes were calculated by the following equation: length × width2/2. All nude mice were sacrificed after treatment, the tumors and heart, liver, spleen, lung, and kidney of nude mice were excised and fixed in 4% formaldehyde over 48 hours, then embedded with paraffin for histopathological analyses.

### 3.10. Statistical analysis

The data were expressed as the mean ± SD. Data were analyzed for statistical significance using a one-way ANOVA, and *p*<.05 indicated a statistically significant difference for all analyses.

## 4. Results and discussion

### 4.1. Preparation and characterization of SOF

SOF was prepared from the self-assembly of compound TAH and CB[8] (molar ratio 1:2) in water. ^1^H NMR characterization of SOF was showed in supporting information. The ^1^H NMR spectra of the 1:2 mixtures of compound TAH and CB[8] in D_2_O (pH = 7.4) displayed broad signals, indicating that supramolecular framework was formed by complexation between the two compounds. The structure of SOF could be stable even after 14 days, while the sizes changed a little after 14 days should be attributed to the slight depolymerization of the SOFs (Fig. S3).When pH decreases to 4.5, the signals of ^1^H NMR spectra became more detailed, indicating that the SOF was unstable at the acidic environment.

**Figure 1.**
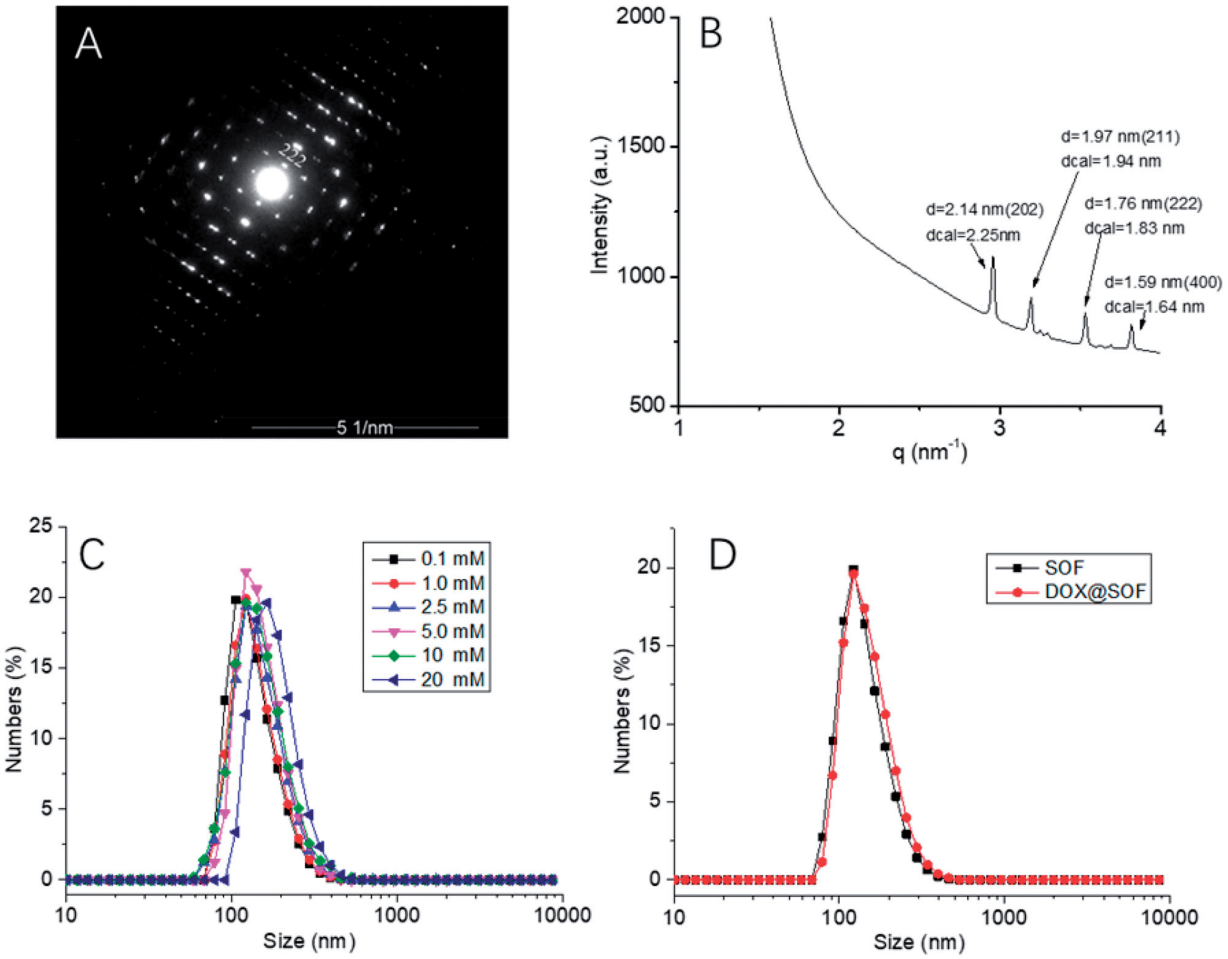
The structural characterization of SOF and DOX release performance of DOX@SOF. (A) SAED image of SOF; (B) small-angle X-ray scattering spectra of SOF structure in solution phase; the hydrodynamic diameter (*D*_H_) of (C) different concentrations of SOF and (D) SOF and DOX@SOF (0.2 mM) determined by DLS.

The sizes of SOF in water at different pH were performed by DLS experiments. The hydrodynamic diameter (*D*_H_) of SOF (0.1 mM) was about 150 nm (Figure 1(C)), and the values of the *D*_H_ were almost the same at different concentrations. After 14 days, the *D*_H_ was about 70 nm (Fig. S4). The zeta potential of SOF was about 44.1 mV. All these results demonstrated the formation of the stable self-assembled framework.

The synchrotron SAXS experiments were performed for determining the periodicity of SOF in aqueous solutions. The SAXS profile of the SOF in water displayed four sharper peaks (Figure 1(B)), which centered around 2.14 nm, 1.97 nm, 1.76 nm, and 1.59 nm. The results matched well with the calculated {202}, {211}, {222}, or {400} spacing. After evaporation of the solution of SOF, microcrystals were afforded. The results were similar to those of SAXS in the aqueous solution. Transmission electron microscopy image revealed the square microcrystals formed. The selected area electron diffraction (SAED) pattern clearly indicated periodicity of 2.1 nm that corresponds to {202} lattice spacings (Figure 1(A)). All these observations confirmed that SOF formed the periodic frameworks in both water and the solid state.

### 4.2. Determination of drug-loading efficiency and *in vitro* release

**Figure 2.**
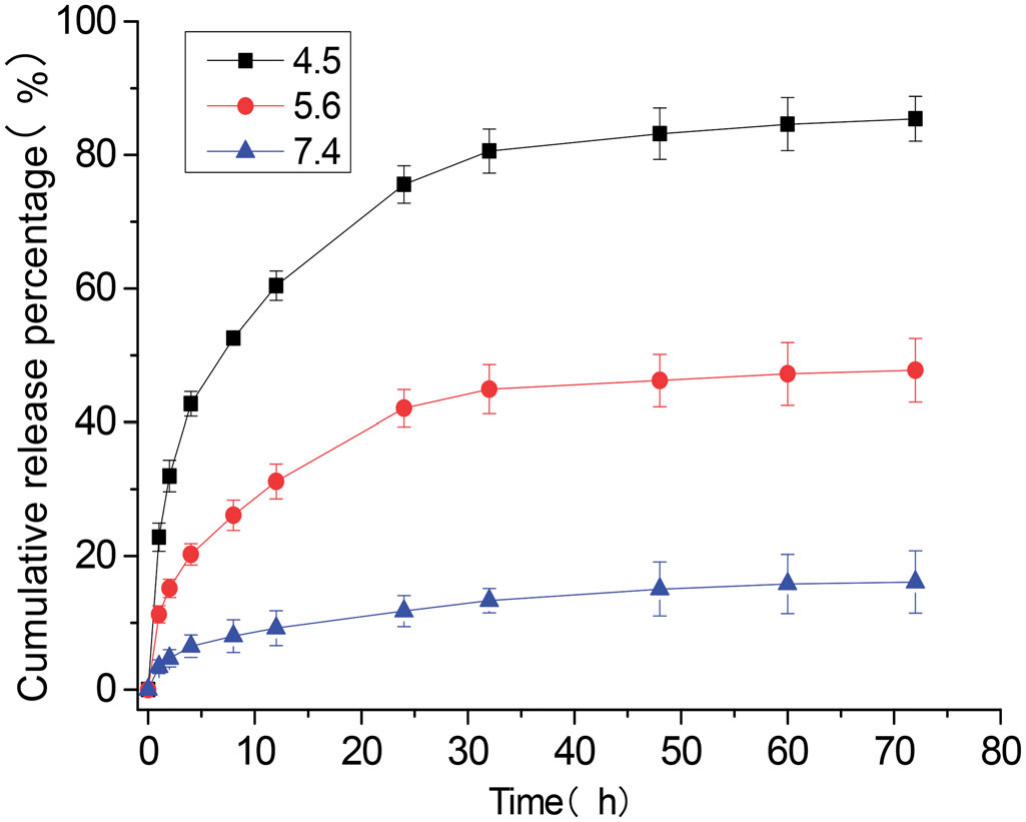
Release of adsorbed DOX from DOX@SOF at 37 °C against time at different pH.

As shown in Figure 1(D), the *D*_H_ of DOX@SOF and SOF was almost the same, illustrating that the DOX was embedded inside the porous of the framework. The drug-loading efficiency and encapsulation efficiency of SOF for different drugs by UV–vis spectroscopy were measured. The results showed that the drug loading efficiency of SOF to DOX was 5.12%±0.14, and the encapsulation efficiency was 81.92%±2.2. As we know, the interaction between the SOF and the drug attenuated sharply under acidic condition, resulting in the release of the drug from SOF. PBS buffer solutions of different pH (4.5, 5.6, and 7.4) were prepared to simulate the normal tissue and tumor microenvironment; the cumulative release curve of the drug is shown in Figure 2. In the pH 7.4 PBS buffer solution, only about 15% of the drug was released from SOF after 72 hours, indicating that SOF could remain large amounts of the drugs without leak within three days in normal plasma circulation, which could greatly reduce the damage of the drugs to normal tissues and promote the drug delivery efficiency during the administration process. About 45% (pH 5.6) and 80% (pH 4.5) of the drugs were released from SOF after 72 hours, confirming the pH-responsiveness of DOX@SOF. The results illustrated that SOF could selectively release drugs in the acidic environment of tumor cells, thereby reduced the release of drugs in normal tissues and enhanced the anti-tumor effects.

### 4.3. Cytotoxicity and apoptosis assays

**Figure 3.**
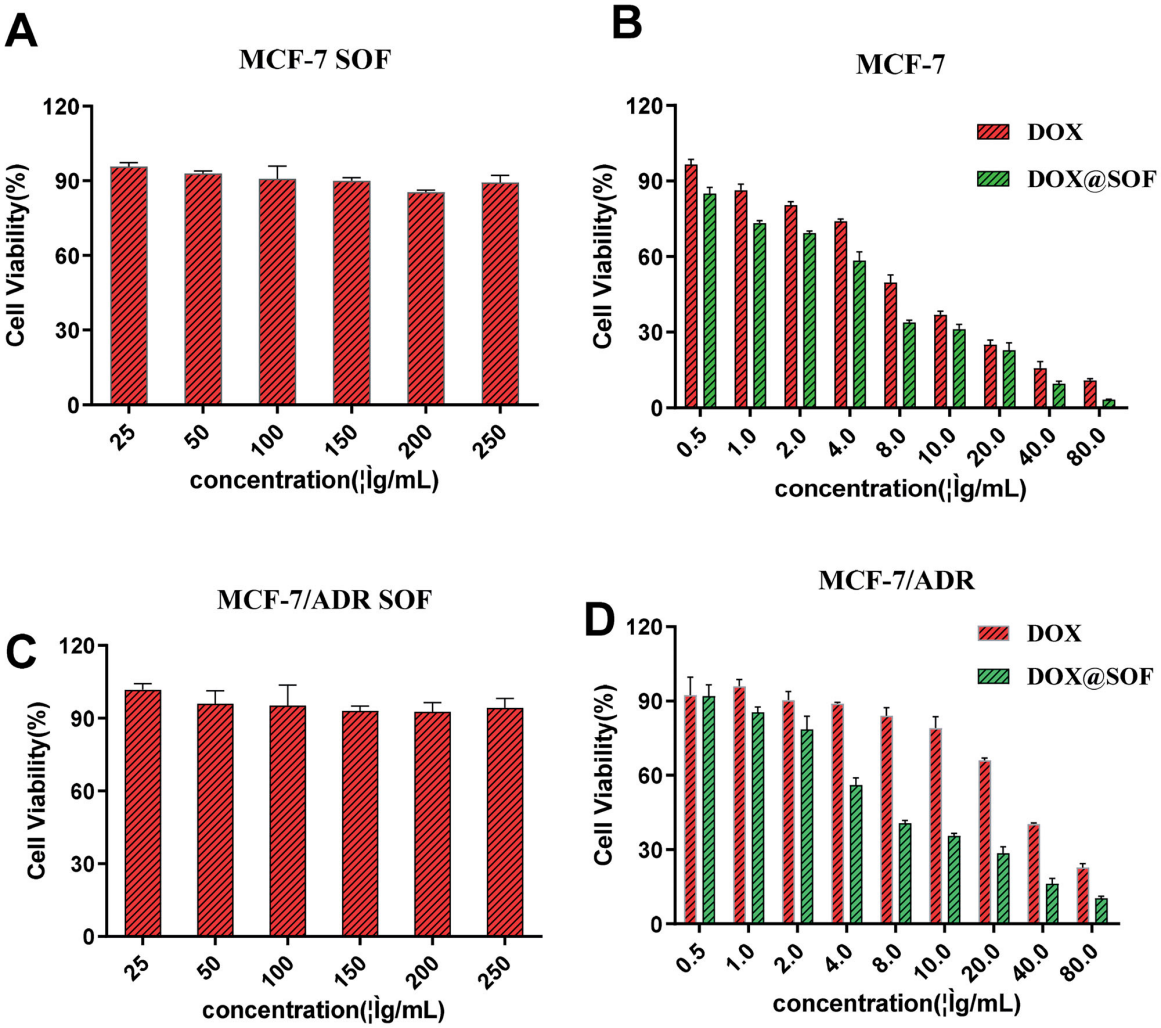
The therapeutic effect of DOX@SOF. Relative viabilities of MCF-7 cells treated with different concentrations of (A) SOF, (B) free DOX and DOX@SOF for 48 h. Relative viabilities of MCF-7/ADR cells treated with different concentrations of (C) SOF, (D) free DOX and DOX@SOF for 48 h (mean ± SD, *n* = 3).

The biocompatibility and treatment effect of SOF *in vitro* were investigated with MCF-7/ADR and MCF-7 cells by CCK-8 method. The survival rate of MCF-7/ADR and MCF-7 cells had above 80% even the concentration of SOF was 250 µg/mL (Figure 3(A,C)), indicating that SOF was low-toxic and suitable as a potential drug carrier. Additionally, for MCF-7/ADR and MCF-7 cells, the cytotoxicity of DOX@SOF was higher than that of free DOX (Figure 3(B,D)), indicating that SOF could increase the cytotoxicity of drug on MCF-7/ADR and MCF-7 cells. It was probably due to an easier cellular uptake of DOX@SOF (with a particle size of about 100 nm). The IC_50_ values of free DOX and DOX@SOF in MCF-7 cells were 6.044 μg/mL and 4.360 μg/mL, respectively. While the IC_50_ values of DOX and DOX@SOF in MCF-7/ADR cells were 30.48 μg/mL and 7.724 μg/mL, respectively. DOX@SOF significantly improved the cytotoxicity of DOX against MCF-7/ADR, reflecting that DOX@SOF could overcome the drug resistance of MCF-7/ADR cells. All these results revealed that DOX@SOF could transport DOX into the cancer cells with high efficiency.

**Figure 4.**
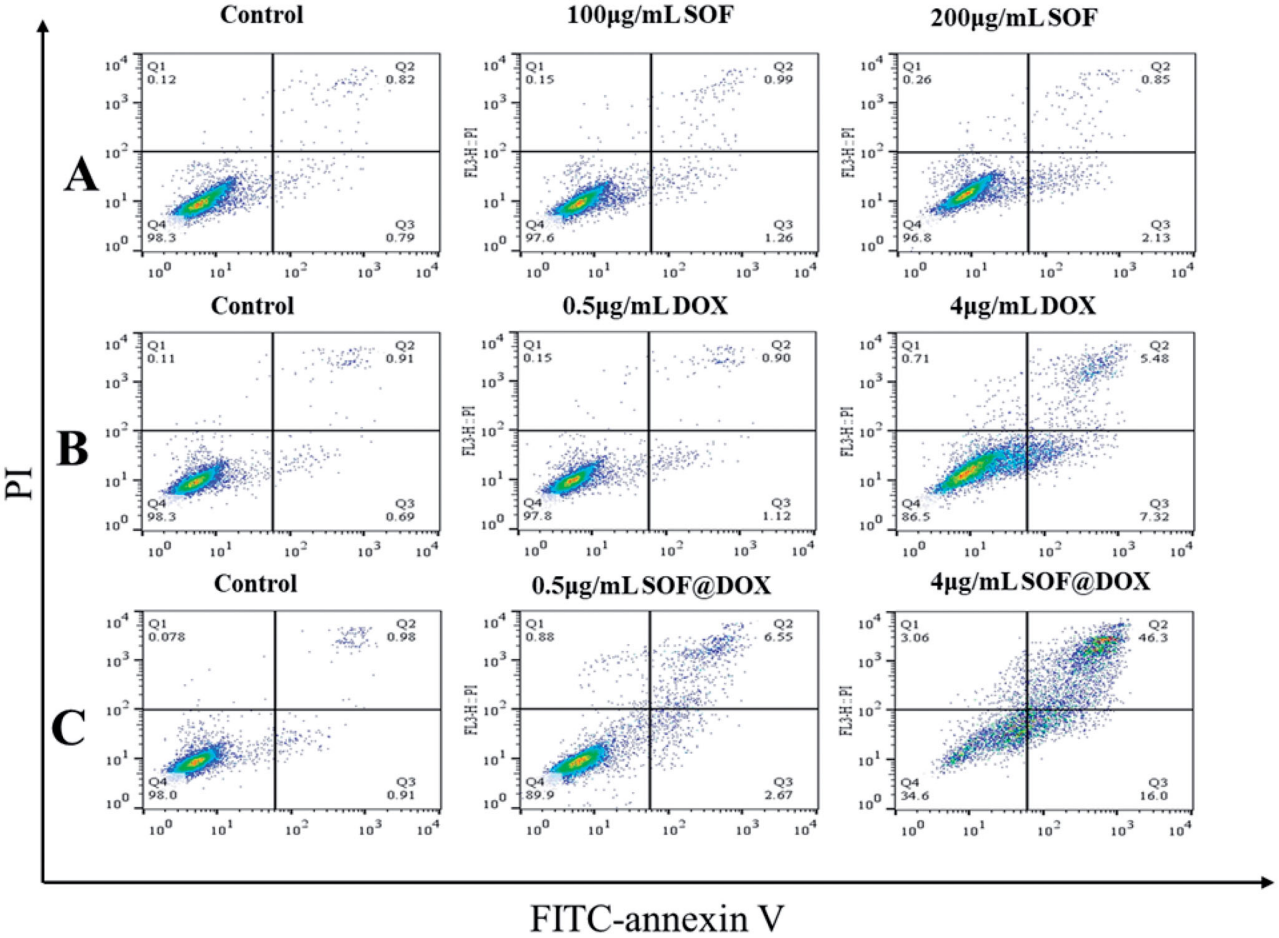
Apoptosis assay of the DOX@SOF *in vitro*. The apoptosis of MCF-7/ADR cells treated with SOF (A), free DOX (B), and DOX@SOF (C) for 48 h.

Furthermore, the apoptosis of MCF-7/ADR cells treated with different materials was detected by flow cytometry. The MCF-7/ADR cells treated with SOF (200 µg/mL) for 48 hours did not show significant apoptosis (Figure 4(A)) and low concentration of free DOX also could not induce apoptosis of MCF-7/ADR cells (Figure 4(B)), indicating the resistance to free DOX of MCF-7/ADR, which was consistent with the experimental results of CCK-8. While, DOX@SOF could induce apoptosis of MCF-7/ADR cells at low concentrations and was dose-dependent (Figure 4(C)). All these results revealed that DOX@SOF could reverse the resistance of MCF-7/ADR cells to DOX and induce apoptosis of MCF-7/ADR cells.

### 4.4. Cellular uptake *in vitro*

**Figure 5.**
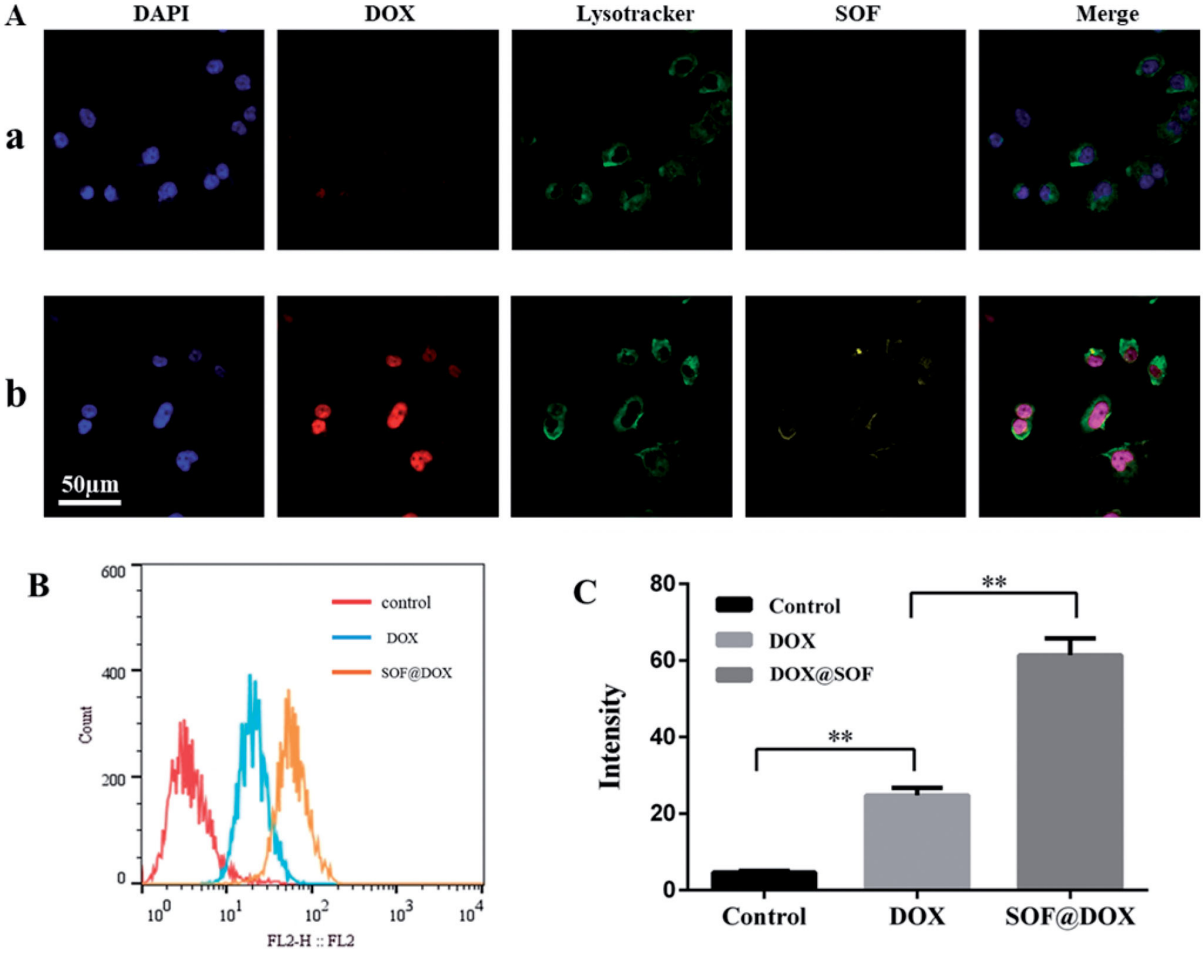
Cellular uptake of DOX@SOF. (A) CLSM images of MCF-7/ADR cells after 6 h incubation with DOX (a) and DOX@SOF (b). (B, C) The fluorescence intensity of MCF-7/ADR cells cultured with culture medium, SOF, and DOX@SOF (mean ± SD, *n* = 3; **p<.01).

One of the important reasons for the failure of chemotherapy is the low internalization of drugs by resistant cells. Therefore, the internalization of DOX@SOF and free DOX by MCF-7/ADR cells was evaluated by flow cytometer and CLSM. After incubation with MCF-7/ADR cells for six hours, the nucleus and lysosome were labeled with DAPI and Lysotracker Green DND-26, respectively. As shown in Figure 5(A), the fluorescence intensity of the DOX@SOF group was significantly higher than that of free DOX, and the fluorescence signal of lysosome stained Lysotracker Green DND-26 overlapped with the fluorescence signal of SOF. Meanwhile, the fluorescence signal of DOX overlapped with the fluorescence signal of the nucleus stained with DAPI, indicating that MCF-7/ADR cells internalized DOX@SOF, and then DOX@SOF released DOX into cytoplasm with the disintegration of lysosome. DOX further entered into the nucleus to kill drug-resistant tumor cells by interfering the activity of chromosomes. To a large extent, the results also indicated that DOX@SOF could effectively reverse the drug resistance to DOX by increasing DOX internalization into MCF-7/ADR cells.

### 4.5. Toxicity and antitumor effects *in vivo*

**Figure 6.**
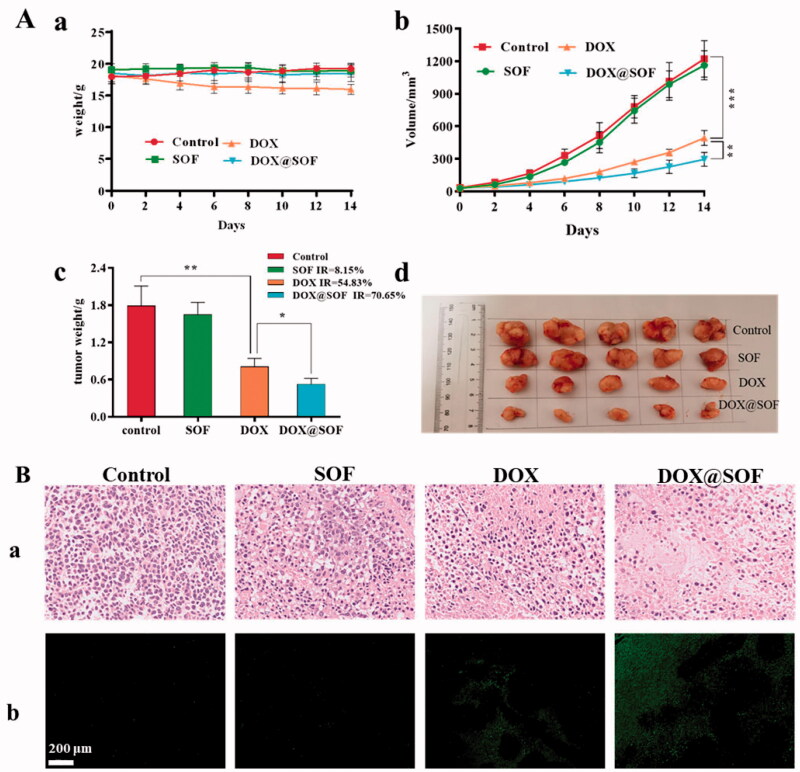
DOX@SOF against the tumor model of BALB/c nude mice. (A) The body weight curve (a), the tumor volume changes in different groups (b), the excised tumor tissues from all groups (c), and the tumor image in different groups (d). (B) The tumor tissue section after different treatment. The image stained by (a) HE (×200), (b) TUNEL (×200) (mean ± SD, *n* = 5, *p<.05, **p<.01, ***p<.001).

In order to study the anti-tumor activity of DOX@SOF *in vivo*, nude mice bearing MCF-7/ADR tumor were established. On the 9th day after inoculation (tumor volume is about 30 mm^3^), the nude mice were randomly divided into four groups randomly: PBS (control), DOX, SOF, and DOX@SOF group. Drugs were injected via the tail vein every other day with a dosage of 4.5 mg/kg (equivalent concentration of DOX). The result is shown in Figure 6(A), during the administration period, the body weights of nude mice had no significant difference except the DOX group, indicating that DOX@SOF reduced the systemic toxicity of DOX. The tumor volume and weight of the DOX@SOF group were the smallest, revealing the MDR was reversed by the enhanced therapeutic activity of DOX@SOF compared with free DOX.

To further evaluate the therapeutic effects, tumor tissues were analyzed by H&E and TUNEL staining after treatment. As shown in Figure 6(B), in the tumor tissues of the control group and SOF group, clear nucleus and regular morphology were observed. In the tumor tissues of the DOX group and the DOX@SOF group, abnormal nuclei and different degrees of tumor tissue necrosis were observed, indicating that DOX@SOF could effectively inhibit tumor growth. TUNEL assays showed similar tumor growth inhibition. These results revealed that DOX@SOF was promising to be explored as pH-responsive drug delivery complex *in vivo* for cancer chemotherapy.

**Figure 7.**
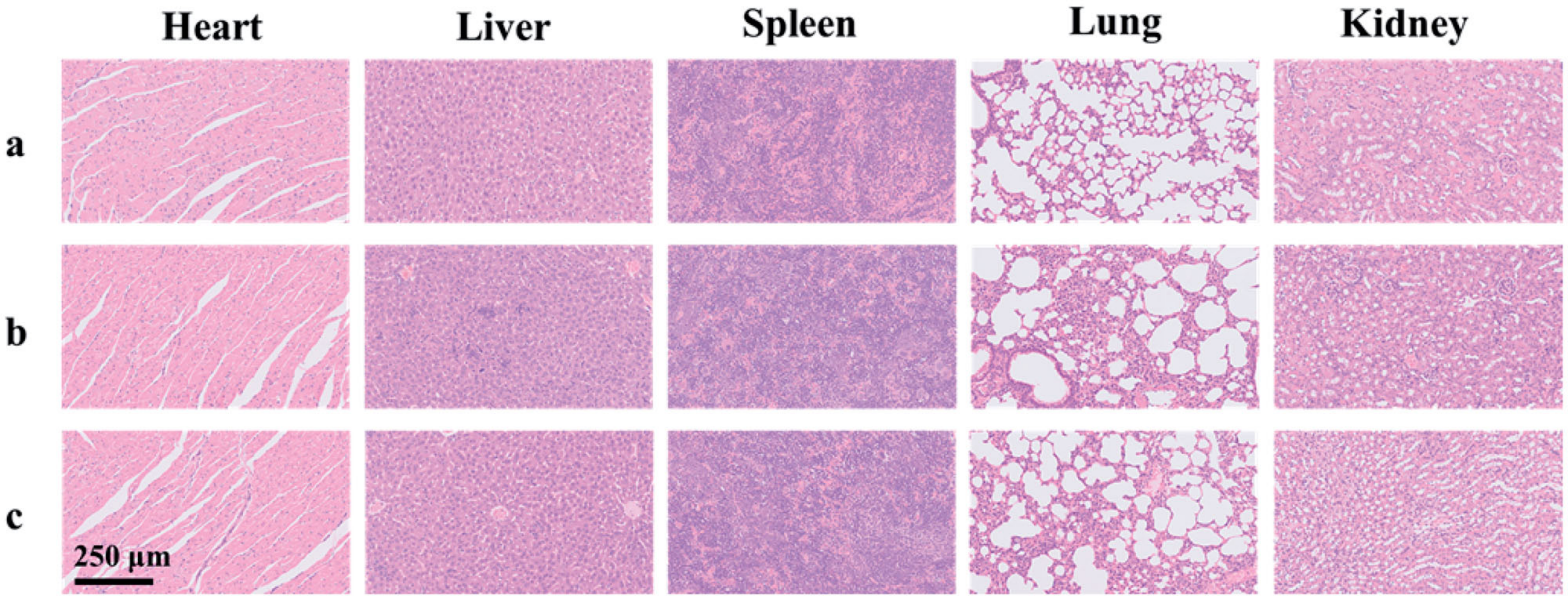
The histological characteristics of the main organs after treatment with glucose solution (a), SOF (b), and DOX@SOF (c) (×200).

The preliminary toxicity of SOF and DOX@SOF in the main organs of nude mice was detected by HE staining. Compared with the normal saline group, the histopathological slice morphology of the major organs of other groups had no significant difference (Figure 7). The results showed that SOF and DOX@SOF had good histocompatibility and no serious adverse reactions for major organs.

## 5. Conclusions

In summary, we have presented a new strategy to fabricate a pH-responsive supramolecular organic framework DDS-DOX@SOF to reverse the MDR of MCF-7/ADR tumor. The DOX@SOF exhibited good biocompatibility, pH regulated drug release, enhanced permeability, and retention effect. At acid condition, the hydrazone bonds would be broken, which result in the dissociation of SOF, and then the drugs would be released from the system. Both *in vitro* and *in vivo* experiments reflected that therapeutic activity of DOX@SOF was significantly enhanced compared with that of free DOX for the MCF-7/ADR tumor cells and tumors. This study provides a new strategy to productively prepare stimulus-responsive supramolecular drug delivery complex for treatment of drug-resistant cancer, presenting inspiring scientific interests in exploring new drug delivery strategy and reversing multi-drug resistance for clinical chemotherapy.

## Supplementary Material

Supplemental Material

## Data Availability

The data that support the findings of this study are available from the corresponding author, F. Yang, upon reasonable request.
